# Evaluation of Tertiary Neuropsychiatry Pilot Service: Pitfalls, Challenges, Outcomes and Success

**DOI:** 10.1192/bjo.2024.98

**Published:** 2024-08-01

**Authors:** Roopa Rudrappa, Kaanthan Jawahar, Nicholas Andrews, Katie French, Mohanbabu Rathnaiah

**Affiliations:** 1 Derbyshire Healthcare NHS Foundation Trust, Derby, United Kingdom; 2 University Hospital Bristol and Weston NHS Foundation Trust, Bristol, United Kingdom; 3 Institute of Mental Health, University of Nottingham, Nottingham, United Kingdom

## Abstract

**Aims:**

Neuropsychiatry, being at the interface between Neurology and Psychiatry, can fulfil the unmet needs of a cohort of people with complex presentations including psychiatry symptoms associated with neurological diseases and atypical psychiatry presentations with possible underlying aberrant brain processes. However, the development and provision of Neuropsychiatry services have lagged behind in the United Kingdom and some parts of the world, at the cost of ongoing symptom burden and reduced quality of life for vulnerable groups of patients. We set up a tertiary pilot service of Neuropsychiatry in Derbyshire from March 2022 and have been successfully operating both outpatient Neuropsychiatry clinics as well as inreach on to psychiatric wards. We set out to evaluate our service and explore the challenges and outcomes associated with our service development.

**Methods:**

A mixed methods evaluation was completed, and the data were extracted from patient records and assessments. Feedback responses were obtained from referring clinicians and service users utilising structured feedback forms for each group. A thematic analysis approach was completed for qualitative responses. More than 140 patients have already been assessed by our Neuropsychiatry service to date, out of which we completed an initial analysis of records of 70 patients referred between March 2022 and February 2023. We further revisited the challenges (lack of resources including clinic space, admin and dedicated electronic medical records (EMR) section).

**Results:**

67% of referrals were from Neurology services with Functional Neurological Disorder (FND) predominating. 74% of patients referred had more than one diagnosis/symptom cluster. Patients reported significant benefits and overall positive experiences from the service. One patient reported, “After 3 years I finally not only have answers to my symptoms but also an explanation as to why. Without this service, I believe I would be still struggling.” Similar positive feedback was obtained from referring clinicians.

**Conclusion:**

Our results demonstrate that a successful tertiary Neuropsychiatry service can be established and run even under challenging circumstances including lack of resources. Our service now has a dedicated clinic running every week, a dedicated EMR section and we are currently in the process of submitting business plans towards sustainable commissioning. Furthermore, our service has been instrumental in reducing the length of inpatient stay, facilitating early discharges, diagnosing and treating reversible conditions that mimicked primary psychiatric issues, as well as improving the quality of life of a vulnerable cohort of people previously diagnosed with complex conditions such as FND and personality disorders.

